# A positive attitude towards provision of end-of-life care may protect against burnout: Burnout and religion in a super-aging society

**DOI:** 10.1371/journal.pone.0202277

**Published:** 2018-08-09

**Authors:** Tsuyoshi Okamura, Masaya Shimmei, Akinori Takase, Shiho Toishiba, Kojun Hayashida, Tatsuya Yumiyama, Yukan Ogawa

**Affiliations:** 1 Research Team for Promoting Independence of the Elderly, Tokyo Metropolitan Institute of Gerontology, Tokyo, Japan; 2 Institute for Future Engineering, Tokyo, Japan; 3 Institute of Regional Development, Taisho University, Tokyo, Japan; 4 Graduate School of Humanities and Social Sciences, University of Tsukuba, Tsukuba, Japan; 5 Department of Buddhist Studies, Taisho University, Tokyo, Japan; 6 Department of Social and Human Sciences, Tokyo Institute of Technology, Tokyo, Japan; Universita degli Studi Di Cagliari, ITALY

## Abstract

**Aim:**

The aim of our study was to investigate factors associated with burnout of nurses and care workers in nursing homes and geriatric hospitals in Japan. The use of Buddhist priests, the major religion in Japan, was also explored.

**Methods:**

Questionnaires for nurses and care workers were sent to 10 care facilities. The survey questions included basic demographic information, the Japanese Burnout Index and the Japanese version of the Frommelt Attitude Toward Care Of Dying Scale Form B. They also asked questions about use of Buddhist priests for tasks such as helping to manage the anxiety or distress of patients, families, and staff, or providing sutra chanting.

**Results:**

In total, 323 questionnaires were returned, of which 260 were used for analysis. Only 18 (6.9%) answered that they had any religious beliefs, which was relatively low compared to 27% from governmental survey data. In total, however, 71% expressed a need for Buddhist priests to help with anxiety or distress among patients. A positive attitude towards providing end-of-life care was a protective factor against depersonalization. It was, however, also related to lower feelings of personal accomplishment.

**Conclusion:**

Care homes and geriatric hospitals may want to consider calling more on religious resources as a support for staff and patients.

## Introduction

In total, 27.1 percent of Japan’s population is now over 65 years old [1], the highest level in the world. This percentage is predicted to continue to grow [2], with projections suggesting that it will reach a steady state of 40% in the year 2055. These figures are because Japan has a low birth rate (an overall fertility rate of 1.5) [3] and low migration rates compared with other Organisation for Economic Co-operation and Development (OECD) countries (0.05% in Japan compared with the OECD average of 0.6%) [4]. Life expectancy is increasing, but the number of deaths in Japan is expected to be twice the number of the births by the year 2030 [5].

Over time, the percentage of people dying in hospitals in Japan has increased from 10% in 1951 up to 80% by around the year 2000 [6]. When nursing home residents were dying, it was not unusual to transport them to hospital for the purpose. Now, however, this trend is being reversed. According to one report, the Japanese government has set up special payments for end-of-life care in nursing homes, and the rate of deaths in nursing homes has risen from 36% in 2010 to 50% in 2013 [6]. Those who care for older people in long-term care facilities may therefore have a new and unfamiliar role in providing end-of-life care.

Figures from the Ministry of Health, Labour and Welfare show that the number of reports of elder abuse in geriatric institutions has risen from 273 in 2006 to 1640 in 2015 [7]. One incident in particular has made headlines in Japan: a former nursing care employee was arrested for allegedly killing three older people in February, 2017 [8]. The reason for this incident is not clear, but the possible role played by burnout has been discussed. Maslach and Leiter suggested that burnout could be split into sub-categories of emotional exhaustion, depersonalization and personal accomplishment. Depersonalization, or cynicism, is described as negative or inappropriate attitudes toward clients, irritability, loss of idealism, and withdrawal [9]. Preventing burnout among the staff of long-term care facilities is an important issue, especially in a super-aging society.

Burnout among healthcare staff in nursing homes has been the subject of previous research. A systematic review by Cooper et al. [10] showed that individual demographic factors such as being married, older, more educated, and having a sense of optimism had a buffering effect on burnout. In Japan, Tanaka et al [11] reported that burnout was related to feelings of criticism and hostility towards residents among long-term care facility employees. To the best of our knowledge, however, relationships between burnout, provision of end-of-life care and religion in long-term care facilities have not been discussed. Religion is reported to have a buffering effect in stressful situations [12,13]. Provision of end-of-life care may involve existential anxiety, so it is rational to include religious factors in the study of burnout in long-term care facilities, as a potential buffering factor.

As a first step in considering religion, we decided to focus on the most popular religion in Japan. The major religions there are Buddhism, Shintoism, and Christianity. The Japanese General Social Survey [14], the largest social survey in Japan, found that 66.7% of respondents who practiced a religion were Buddhists, compared with 2.3% who practiced Shintoism and 3.3% Christianity. A survey conducted by the Ministry of Education, Culture, Sports, Science and Technology Institute [15] found that there were 340,000 Buddhist priests in Japan, compared with 80,000 Shinto and 30,000 Christian. These data show that Buddhism is the most popular religion in Japan.

The aim of this paper is to investigate factors associated with burnout of nurses and care workers in such facilities. It also explores the use of Buddhist priests, the major religion in Japan.

## Materials and methods

### Participants

The anonymous questionnaire survey was conducted between May and July 2017. Two hospitals and eight nursing homes were included in the study, selected by convenience sampling. They were all located in or near the Tokyo metropolitan area, and run by secular medical or welfare corporations. We asked the directors of the facilities about the number of staff providing care for patients at each location, then delivered that number of questionnaires to them. We asked the staff to complete the questionnaire and return it to a box with a window too small to permit later removal. We asked the directors not to require staff to answer or to check whether they had done so. The last page of the questionnaire included vouchers equivalent to about five dollars. The staff could use the voucher regardless of whether they returned the questionnaire. Because our study was anonymous questionnaire survey, returning of the questionnaire was regarded as consent.

A total of 323 questionnaires out of 338 delivered were retrieved (valid response rate = 96%). The majority of respondents were nurses (n = 82, 25%) and certified care workers (n = 179, 55%). A certified care worker holds a national license under the Certified Social Worker and Certified Care Worker Act, and has passed the certified care worker examination on knowledge and skills of physical care. Certified care workers can perform mucus aspiration. According to the Ministry of Health, Labour and Welfare, there are around 1,500,000 certified care workers in Japan [16]. The others included 11 occupational therapists, five doctors, five care-planners, and four psychologists. Only responses from nurses and certified care workers were included in the analysis because it was felt that nurses and certified care workers are likely to be closest to the patients or users of such facilities. It was also considered best to avoid heterogeneity of participants.

The study protocol was approved by the Ethics Committee of the Taisho University (17–001).

### Measures

The questionnaire included sociodemographic variables such as sex, age group, profession, and length of time working in long-term care facilities, including other facilities. The participants were also asked whether they held any religious beliefs.

Burnout was assessed using the Japanese Burnout Index [17]. This is a 17-item, five-scale measure that assesses burnout, adapted to fit the Japanese social context. It consists of three subscales, emotional exhaustion (five items), depersonalization (six items), and personal accomplishment (six items). Higher scores for “emotional exhaustion” and “depersonalization” indicate more likelihood of emotional exhaustion and depersonalization, which are related to increased burnout. A higher score for “personal accomplishment” shows a stronger feeling of personal accomplishment, which is related to lower levels of burnout. This factor structure is similar to the Maslach Burnout Inventory, which is considered the best measurement of burnout [18].

The Japanese version of the Frommelt Attitude Toward Care Of Dying Scale Form B (FAT-COD-Form B-J) [19] was used to measure attitude towards provision of end-of-life care. The full form is a 30-item, five-scale measure, but we used the shorter form. This consists of two subscales, one on positive attitude towards providing end-of-life care (three items) and one on perceptions of patient- and family-centered care (three items) [20]. A positive attitude towards providing end-of-life care was assessed using items such as “caring for the dying is a value for the respondent”, with five possible answers: “strongly disagree” (1 point), “disagree” (2 points), “neither agree or disagree” (3 points), “agree” (4 points) and “strongly agree” (5 points). Perceptions of patient- and family-centered care were assessed using items such as “family should be involved in the physical care of the dying”, with the same five possible answers. We also asked whether the participants had personal experience of end-of-life care at home, or during the previous year at work.

Use of the Buddhist priest was assessed by asking about whether participants felt the need for a priest in four cases: 1) To help with the anxiety or distress of the patients/users, 2) To help with the anxiety or distress of family of the patients/users, and 3) To help with the anxiety or distress of staff of the facilities, and 4) To provide sermons or sutra chanting. Sutra chanting is the reading out of sacred texts, and is very common in Buddhist rituals such as funeral and/or memorial services, and festivals celebrating the growth of children. It is usually performed by Buddhist priests, but attendees are sometimes encouraged to join in during some parts of the ritual. Most people are no longer familiar with Buddhist teachings and doctrine, so few attendees will understand exactly what is chanted. They do, however, know that the chanting represents something good and virtuous, from the meaning of the Chinese characters. Several studies have reported that sutra chanting has a favorable effect on physical and mental health [21,22], but this is not fully established.

The participants were asked to choose from “yes”, “don’t know”, and “no”. Those who answered “don’t know”and “no” were considered to have no desire to see the priest.

### Statistical analysis

Student’s t-test was used for comparison of continuous variables (i.e., average scores on the psychometric measures). The association between burnout and attitude towards providing end-of-life care was initially analyzed using Pearson correlation analysis. Multivariate analyses were then performed using length of time working in long-term care facilities (under 10 years / over 10 years), institutional type (hospitals / nursing homes), and having religious beliefs as control variables. A p-value of 0.05 was regarded as statistically significant. Analyses were performed using IBM SPSS 23 (IBM Corp).

## Results

The characteristics of the study participants are shown in Table 1. Of the 261 valid responses, only 18 (6.9%) answered that they had religious beliefs.

**Table 1:** Characteristics of the 261 study participants.

Characteristics		number	(%)
sex			
	male	78	(30)
	female	183	(70)
age			
	20's	61	(23)
	30's	70	(27)
	40's	64	(25)
	50's	53	(20)
	60's and over	13	(5)
length of the career			
	under 10 years	121	(46)
	10 years and over	140	(54)
profession			
	nurses	82	(31)
	care workers	179	(69)
institutions			
	hospitals	76	(29)
	nursing homes	184	(71)
Having religion			
	yes	18	(7)
	no	242	(93)

The nurses’ and care workers’ desire to see the Buddhist priest are shown in Table 2.

**Table 2:** The proportion of nurses and care workers in long-term care facilities who wanted support from Buddhist priests for particular aspects of care.

To help to manage the anxiety or distress of patients	71%	(183/259)
To help to manage the anxiety or distress of families	66%	(172/260)
To help to manage the anxiety or distress of staff	42%	(107/259)
Sutra chanting	47%	(120/256)

There were some differences in the findings on burnout by experience and location. Experienced staff tended to show less emotional exhaustion, and staff in nursing homes were more likely to show depersonalization. Those who answered yes to needing religious care had a statistically significant lower score for personal accomplishment. Those who favored sutra chanting in the facility, however, had lower scores for emotional exhaustion. The average scores for the Japanese Burnout Index by subgroup are shown in Table 3.

**Table 3:** The average score for the Japanese Burnout Index for each characteristics of the participants.

		emotional exhaustion		de- personalization		personal accomplishment	
**Basic information**							
Sex							
	male	3.0±0.9		2.2±0.8		3.4±0.8	
	female	3.1±0.9		2.2±0.8		3.4±0.8	
Age							
	under 50	3.1±0.9		2.2±0.8		3.4±0.8	
	50 and over	2.8±0.8	*	2.0±0.6	*	3.5±0.7	
Length of time working in long-term care facilities							
	under 10 years	3.2±0.9		2.3±0.8		3.4±0.8	
	over 10 years	2.9±0.9	*	2.1±0.7		3.4±0.8	
Profession							
	nurses	3.0±0.8		2.1±0.7		3.4±0.8	
	care workers	3.1±0.9		2.2±0.8		3.4±0.8	
Institutions							
	hospitals	3.0±0.8		2.0±0.6		3.5±0.7	
	nursing homes	3.1±0.9		2.2±0.8	*	3.4±0.8	
Religious belief							
	present	2.8±0.9		2.2±0.8		3.0±0.7	
	absent	3.0±0.9		2.2±0.8		3.5±0.8	*
**Experience of end of life care**							
Personal experience of end of life care							
	present	3.0±0.8		2.1±0.7		3.3±0.8	
	absent	3.1±0.9		2.2±0.8		3.5±0.8	
Experience of end of life care at work							
	present	3.1±0.9		2.2±0.7		3.4±0.8	
	absent	2.8±0.8	*	2.1±0.6		3.4±0.8	
**Needs for Buddhist priests**							
To help to manage the anxiety or distress of patients							
	present	3.1±0.9		2.2±0.8		3.3±0.8	
	absent	3.0±0.9		2.2±0.8		3.6±0.8	**
To help to manage the anxiety or distress of families							
	present	3.1±0.9		2.2±0.7		3.3±0.8	
	absent	3.0±0.9		2.1±0.8		3.6±0.8	*
To help to manage the anxiety or distress of staff							
	present	3.1±0.9		2.2±0.7		3.2±0.8	
	absent	3.0±0.8		2.1±0.8		3.6±0.7	***
Sutra chanting							
	present	2.9±0.9		2.1±0.7		3.3±0.8	
	absent	3.2±0.9	*	2.2±0.8		3.5±0.9	*
							

“emotional exhaustion” ranges 5 to 25; “de-personalization” ranges 6 to 30; “personal accomplishment” ranges 6 to 30.

* p<0.05;

** p<0.005;

*** p<0.001

Attitudes towards provision of end-of-life care among the nurses and care workers are shown in Table 4. Those with religious beliefs showed a more positive attitude towards this type of care, as did older nurses and care workers.

**Table 4:** Average scores of the Frommelt Attitude Toward Care Of Dying Scale Form B for each characteristics of the participants.

		Positive attitude toward caring for the dying		Perception of patient- and family- entered care
Sex				
	male	10.9±2.1		11.1±1.9
	female	10.9±1.8		11.6±2.0
Age				
	under 50	10.8±1.9		11.4±2.0
	50 and over	10.9±1.8		11.6±2.0
Length of time working in long-term care facilities				
	under 10 years	10.6±2.0		11.4±2.0
	over 10 years	11.7±1.8	*	11.4±2.0
Profession				
	nurses	10.9±1.9		11.7±1.8
	care workers	10.8±1.9		11.3±2.1
Institutions				
	hospitals	10.7±2.0		11.4±1.9
	nursing homes	10.9±1.9		11.5±2.0
Religious belief				
	present	12.4±2.1		11.3±1.8
	absent	10.7±1.8	***	11.5±2.0
				

“Positive attitude toward caring for the dying” ranges 3 to 15; “Perception of patient- and family- entered care” ranges 3 to 15.

Last, the association between burnout and attitude towards providing end-of-life care is shown in Table 5. A positive attitude towards providing end-of-life care appears to be a protective factor against depersonalization, but was also related to lower personal accomplishment.

**Table 5:** Association between burnout and attitude towards provision of end-of-life care.

	Bivariete analysis		Multivariate analysis	
	Pearson's correlation coefficient	p-value	Partial correlation coefficient	p-value
emotional exhaustion *positive attitude toward caring for the dying	-0.150	p = 0.016	-0.121	0.053
depersonalisation *positive attitude toward caring for the dying	-0.217	p<0.001	-0.219	<0.001
personal accomplishment *positive attitude toward caring for the dying	-0.231	p<0.001	-0.199	0.001
emotional exhaustion *perception of patient- and family- entered care	0.002	p = 0.973	-0.002	0.975
depersonalisation *perception of patient- and family- entered care	0.041	p = 0.516	0.035	0.583
personal accomplishment *perception of patient- and family- entered care	-0.175	p = 0.005	-0.177	0.005

* p<0.05

** p<0.005

*** p<0.001

## Discussion

Our results suggest that a positive attitude towards providing end-of-life care is a protective factor against depersonalization. We also found considerable desire for religious intervention by Buddhist priests in long-term care facilities. These results are very helpful in the current Japanese context of an aging society. If the increasing number of deaths in nursing care facilities was a burden on the staff, it is rational that a positive attitude would protect against burnout. A positive attitude may also relate to high goals, however, which explains why it was also linked to a feeling of lower personal accomplishment. As the number of older people increases, and more have incurable diseases such as dementia, severe frailty, and terminal cancer, the burden on nurses and care workers in long-term care facilities will be heavier. This trend is likely to continue as the supply and demand gap for care workers is estimated to reach 380,000 in 2025, according to the Ministry of Health, Labour and Welfare [23].

To support care professionals providing end-of-life care, the study indicated a potential role for religion. However, religious needs in long-term care facilities have mainly been considered in the context with spiritual needs. Ødbehr et al [24] indicated that spiritual needs included 1) the need for serenity and inner peace, 2) the need for confirmation (or affirmation, added by the authors), and 3) the need to express faith and beliefs. However, the use of terms maybe culturally sensitive. Takeda [25], for example, stated that the concept of spirituality is not established in Japan, and there is no everyday word with that meaning.

For this reason, instead of asking about spiritual needs, we asked about respondents’ the needs for specific actions by Buddhist priests. Governmental survey data [26] suggest that 27% of Japanese people had religious views. This percentage was smaller in our study, but expressed desire to see Buddhist priests was greater, indicating that there may be a gap between belief and practice. One explanation for this may be that staff in care facilities are so heavily burdened that any help is welcomed. Another is that Buddhist priests’ religious behaviors such as sutra chanting have become so habitual to Japanese people that they are not regarded as religious activities.

The role of religion in the care of older people has seldom been discussed in Japan. However, Buddhism has a long history in the country, and plenty of resources including 77,000 temples [15], 340,000 priests [15], and 168 organizations [15]. Buddhist priests spend time training to support people in distress. They may therefore be an additional resource that could be usefully incorporated into the care system. Future studies should assess the effect of interventions from Buddhist priests in the health care system. For example, we are planning a study on the effect of having Buddhist priests visit long-term care facilities more regularly and provide counseling, and particularly how this affects staff turnover.

### Limitation

Our study had some limitations. First, the institutions included may not be nationally representative, so the results may not be generalizable. Our study did not use random sampling, so the results may overestimate the preference for religious involvement in care, in that the institutions that were involved may not have high barriers to such new ideas. Second, the practical constraints meant we were unable to include many sociodemographic items. Marital status, household information, educational attainment, and socioeconomic status are often included in studies of this kind, and might have provided useful comparative information. Third, the number of participants who expressed religious views was very small compared with the national average, and this might have affected the results. Fourth, a cross-sectional design has inherent limitations in determining any causal relationships. Fifth, use of survey incentive (vouchers equivalent to about five dollars) may have caused some bias.

## Supporting information

S1 Data (SAV)
